# A Neglected Communication

**Published:** 1867-04

**Authors:** 


### A NEGLECTED COMMUNICATION.

We find among our papers an article entitled: "Number of teeth filled where the nerves have been destroyed, or were already dead, and the time when filled, commencing with April 26, 1864, and ending December 31, 1865."

The report goes minutely into detail, giving the patient's initials in each case, the particular tooth, and how filled. This we find too tedious for publication, but will gladly publish the annexed summary, furnished by the writer. No name is appended to the paper, but, if our memory is not treacherous, its author is Dr. Benedict, of Detroit, and it was read before the Michigan State Dental Society, January, 1866:

| | |
|---|---|
| Whole number reported in twenty months | 77 |
| Number filled with gold | 55 |
| " " " Wood's metal | 9 |
| " " " tin | 6 |
| " " " Lawrence's amalgam | 2 |
| " " " amalgam of silver and tin | 5 |

CLASSES OF TEETH.

| | |
|---|---|
| Inferior anterior molars | 13 |
| " posterior " | 1 |
| " " bicuspids | 4 |
| " anterior " | 2 |
| Superior posterior molars | 4 |
| " anterior " | 9 |
| " posterior bicuspids | 8 |
| " anterior " | 15 |
| " " cuspidati | 4 |
| " lateral incisors | 7 |
| " central incisors | 9 |
| Not marked | 1 |
| On left side of the mouth | 41 |
| " right " " " | 35 |

Some others have been treated and filled which I have not put down in this report; but none, as far as I know of those not reported, have given any trouble since being filled. Of those reported, a few have had slight touches of dental periostitis; but some have recovered without any medical assistance, while others have readily yielded to the application of iodine, cooling medicines and warm bath for the feet. And so far as I am aware, all are still retained as useful members in their respective dental arches. Some few have quite interesting histories, some of which I will relate:

One filled on the 10th of November, 1864, for a young lady of fifteen or sixteen years, was the left superior anterior molar, decayed on the anterior approximal side. In excavating the cavity, I found the nerve slightly exposed, but did not wound it. It was in the early part of summer. I capped it with Hill's stopping, and filled with *os artificiel*, in hopes to save the nerve alive. I had just commenced to repair her teeth, and expected to be some time in doing it, as they were very bad, and she could not come regularly as she attended school. I therefore let the temporary filling remain, until about through with the rest, when I removed it, and attempted to fill with gold; but in doing so, my assistant happened to strike when I was not quite ready, and drove the instrument into the nerve. I then applied arsenious acid to kill it, and proceeded in my usual way to remove the dead nerve, which I accomplished, except in the anterior buccal fang. I then took a small reamer of Dr. Palmer's pattern, and commenced to enlarge the nerve canal in that fang; took particular pains, as I supposed, to have it follow the canal, but before it had gone over two lines it appeared to be through the root into the flesh. Whether it cut through the side of the fang, or whether through some freak the root was not fully formed, I was not able to tell. I could not find any root or any place where the instrument had left the nerve canal. I applied a pledget of cotton with creosote, and next day, on removal, a thin, bloody, watery fluid flowed quite freely. I then applied the creosote, with a little iodine. She was gone several days, and when I saw her next, pus had formed and discharged through the gum, about two lines above its margin. I probed through the opening, but found no root above where the point of the instrument appeared to leave it. I syringed it out, and applied creosote and iodine again. In a few days, the gum healed, and I carefully removed the cotton from the fang, took a flat bur and enlarged the lower part of the canal to three times its size, about a line and a half in depth, then fitted a piece of lead the size of the bur, with a point fitting the nerve canal, allowing it to extend up the fang as far as it could, without coming in contact with the flesh. I then pressed the lead into the recess made with the bur with a burnisher until it was packed tight against the walls. I then filled the other fangs and crown with gold, and so far it appears as solid and firm as any tooth.

Another that I filled for a Mrs. S., on the 23d of August last, has some peculiarities about it. It was a right central. In February last, she had applied to a Dentist to have her teeth fixed, the centrals being decayed on the approximal surface, but not to the nerve. He put a wedge between the teeth to separate them, and appointed a day to fill, but she being sick, did not come for ten or twelve days, when he cleaned and filled them, the right one being very sore. In about six weeks it suppurated, and she had it lanced, and from that time until I saw her, which was the first week in May, it had been leeched and lanced several times, but nothing else done to effect a cure. I found the gum much inflamed, and raised up over the root of the tooth was a bunch of about three lines in diameter, showing pus just under the mucous membrane. I lanced deeply, and probed to the apex of the root, took out portions of the alveolus, syringed thoroughly, first with castile soap and water, and afterwards with water, then injected creosote and iodine, and placed a pledget of cotton, wet in a solution of iodine and glycerin in the wound, to remain until the next dressing. I drilled into the nerve cavity from the underside of the tooth, and found the nerve dead and mostly gone, and the canal nearly dry. I had the misfortune to break off the point of an instrument in the root, up near the apex, and could not readily get it out; so I applied iodine on cotton, in hopes to rust it out, and continued the application of creosote and iodine, three or four times, in its full strength, each time washing the sore out before applying; after which, I made a weak solution of creosote, iodine and glycerin, and after washing it out with water, would force it up about the root with a syringe, and then apply a pledget of cotton wet in a solution of iodine and glycerin in the mouth of the wound, to keep the air from it. After some drawbacks, on account of sickness of the patient and absence from the city, I found, on the 23d of August, the gum all healed, and to all appearance healthy; and having succeeded in removing the broken instrument, I filled the nerve cavity and dismissed her. Two months after, I saw her. She said it was all right, except it did not feel quite so strong as the other. Histories of others could be given; but I am making this paper too long already. But it shows that heretofore too many teeth have been sacrificed, and the forceps used too much. And we still see the advertisements in the papers, "teeth extracted without pain," holding out inducements to those who know no better, to sacrifice a tooth when it aches a little; yet we would not recommend every one who styles himself "Dentist" to undertake to treat such teeth until he has studied them thoroughly, and has a little more knowledge of the healing art.

